# Acute kidney injury in cancer patients receiving anti-vascular endothelial growth factor monoclonal antibody vs. immune checkpoint inhibitors: a retrospective real-world study

**DOI:** 10.1186/s12885-024-12540-y

**Published:** 2024-06-24

**Authors:** Jianfen Zhu, Xiaokai Ding, Jianna Zhang, Bo Chen, Xiaohan You, Xinxin Chen, Tianxin Chen

**Affiliations:** 1 Department of Internal Medicine Nursing and Endoscopy Center, The First Affiliated Hospital of Wenzhou Medical University, Wenzhou, China; 2 Department of Nephrology, The First Affiliated Hospital of Wenzhou Medical University, Wenzhou, China; 3 Key Laboratory of Intelligent Treatment and Life Support for Critical Diseases of Zhejiang Province, The First Affiliated Hospital of Wenzhou Medical University, Wenzhou, China

**Keywords:** Anti-vascular endothelial growth factor monoclonal antibody (anti-VEGF), Immune checkpoint inhibitors (ICIs), Acute kidney injury (AKI).

## Abstract

**Background:**

Anti-vascular endothelial growth factor monoclonal antibody (anti-VEGF) or immune checkpoint inhibitors (ICIs) combined with chemotherapy are commonly administered to cancer patients. Although cancer patients receiving anti-VEGF or ICIs have been reported to experience an increased risk of acute kidney injury (AKI), comparative studies on the AKI incidence have not been evaluated.

**Methods:**

Cancer patients receiving anti-VEGF or ICIs were retrospectively selected from the hospital information system of the First Affiliated Hospital of Wenzhou Medical University between Jan, 2020 and Dec, 2022 and were divided into two groups according to the treatment regimen: anti-VEGF group and ICIs group. The baseline characteristics were propensity-score matched. The primary outcome was sustained AKI. A comparison of cumulative incidence of sustained AKI was performed by Kaplan-Meier curves and log-rank test. Risks for outcomes were assessed using Cox proportional regression.

**Results:**

A total of 1581 cancer patients receiving anti-VEGF (*n* = 696) or ICIs (*n* = 885) were included in the primary analysis. The ICIs group had a higher cumulative incidence of sustained AKI within one year than the anti-VEGF group (26.8% vs. 17.8%, *P* < 0.001). Among 1392 propensity score matched patients, ICIs therapy (*n* = 696) was associated with an increased risk of sustained AKI events in the entire population (HR 2.0; 95%CI 1.3 to 2.5; *P* = 0.001) and especially in those with genitourinary cancer (HR 4.2; 95%CI 1.3 to 13.2; *P* = 0.015). Baseline serum albumin level (> 35 g/l) was an important risk factor for a lower incidence of sustained AKI in the anti-VEGF group (HR 0.5; 95%CI 0.3 to 0.9; *P* = 0.027) and the ICIs group (HR 0.3; 95%CI 0.2 to 0.5; *P* < 0.001).

**Conclusions:**

Among cancer patients in this real-world study, treatment with ICIs increased incidence of sustained AKI in one year. Baseline serum albumin level was an important risk factor for sustained AKI. The risk factors for sustained AKI differed between the anti-VEGF group and the ICIs group.

**Trial Registration:**

The study has been registered at ClinicalTrials.gov (NCT06119347) on 11/06/2023.

**Supplementary Information:**

The online version contains supplementary material available at 10.1186/s12885-024-12540-y.

## Introduction

Anti-vascular endothelial growth factor monoclonal antibody (anti-VEGF) or immune checkpoint inhibitors (ICIs) combined with chemotherapy have been approved for advanced cancer because of their substantial improvements in survival compared with chemotherapy alone [1–6]. Although combination therapy is effective in prolonging the overall survival of cancer patients, it is necessary to optimize drug selection for patients’ long-term quality of life considering the potential adverse effects of chemotherapy. Drug nephrotoxicity is particularly important because the kidney is one of the most vulnerable organs. Anti-VEGF and ICIs may increase the risk of acute kidney injury (AKI) [7–10]. However, no prior studies directly comparing anti-VEGF vs. ICIs have been published with adverse events of AKI as the primary end point. As a result, the long-term adverse events of AKI between cancer patients receiving anti-VEGF and ICIs remains unclear. To address this question, this large real-world cohort study was designed to compare the AKI events among cancer patients receiving anti-VEGF vs. ICIs. The primary aim of this study was to determine whether the choice between anti-VEGF and ICIs affects the incidence of AKI and to find the risk factors of AKI in these patients.

## Materials and methods

### Study design and setting

This study was a retrospective cohort analysis of patients with malignancies treated with anti-VEGF or ICIs between Jan 2020 and December 2022. The primary event of sustained AKI in cancer patients receiving anti-VEGF (Bevacizumab) was compared with that of the patients with ICIs (Pembrolizumab, Sintilimab, Toripalimab, Camrelizumab or Tislelizumab). The study was approved by the Ethics Committee (Issuing Number KY2023-R206) of the First Affiliated Hospital of Wenzhou Medical University. Trial Registration: The study was registered at ClinicalTrials.gov (NCT06119347) on 11/06/2023.

### Data sources

The hospital information system (HIS) of the First Affiliated Hospital of Wenzhou Medical University contains over 6 million longitudinal patient records, and includes over 50 million follow-up records from 2004 to 2022. The following variables related to patient demographics and therapy administration were collected: age, sex, cancer type, Anti-VEGF and ICIs type and dose, and therapy start and end dates. The first day of patients receiving Anti-VEGF or ICIs was defined as index date. The following baseline data were abstracted from the HIS of our hospital: baseline characteristics and serum creatinine (Scr) data, comorbidities that may influence the development of AKI (chronic kidney disease, hypertension, diabetes, hypovolemia, infections), potential nephrotoxic medications received while on ICIs therapy, and the last date of available follow-up. A computer algorithm was used to obtain baseline Scr (i.e., the measurement obtained closest to, but prior to the date of first anti-VEGF or ICIs dose). The algorithm also provided peak Scr values during the 365-day period following the first anti-VEGF or ICIs dose. All Scr data obtained from the algorithm were then reviewed manually and verified.

### Participants’ inclusion and exclusion

Participants’ inclusion criteria included age ≥ 20 year, a diagnosis of cancer within 12 months before the index date (In situ biopsy was not required, since patients may receive a diagnosis from a biopsy of a metastatic site), receiving Anti-VEGF or ICIs therapy, index date between Jan 2020 and December 2022. Exclusion criteria included patients without valid data, with less than 3 months follow-up, without baseline Scr value within 12 months before the index date, with combination therapy of Anti-VEGF and ICIs, and with chronic renal failure.

### Definition

AKI was defined as an elevation of ≥ 1.5 times baseline Scr according to the Kidney Disease Improving Global Outcomes (KDIGO) Scr criteria [11]. Sustained AKI means the Scr remains ≥ 1.5 times the baseline for at least 72 h [7]. The severity of AKI was defined by the KDIGO staging criteria as follows: Stage 1, Scr increase to 1.5 ~ 2 fold of baseline; Stage 2, Scr increase to 2 ~ 3 fold of baseline; and Stage 3, Scr increase to ≥ 3 fold of baseline or an absolute increase of ≥ 4.0 mg/dl or initiation of renal replacement therapy [11].

### Study outcome

The primary endpoint was the time to the first occurrence of the event endpoint of sustained AKI.

### Statistical analysis

Propensity score matching was used to balance the difference in baseline characteristics between patients who received Anti-VEGF versus those who received ICIs. One-to-one nearest neighborhood caliper matching was used to match patients based on the logit of the propensity score using a caliper equal to 0.2 of the standard deviation of the logit of the propensity score. The standardized mean difference (SMD) was used to assess the balance of each baseline covariate between the groups before and after propensity-score matching. SMDs were defined as follows: 0.2, small; 0.5, medium; and 0.8, large. Continuous variables with a normal distribution were expressed as means ± standard deviation (SD) and compared using Student’s t-test. Categorical variables were expressed by proportions and tested using the chi-square test. Those with skewed distribution were expressed as median and IQR and tested using the Mann-Whitney U-test. The incidence of the primary outcome was expressed using cumulative incidence functions. Comparison of the primary outcome was performed by Kaplan-Meier curves and log-rank test. Cox proportional hazard regression models were performed to estimate hazard ratios and confidence intervals. To test for heterogeneity by subgroups, the Cox models were adjusted to an interaction term of the two groups with the baseline subgroups. *P* < 0.05 was considered statistically significant. Data were analyzed using the SPSS version 22 (SPSS, Inc., USA) or R version 4.2.1 (R Core Team (2022)).

## Results

### Patient characteristics

**Fig. 1:**
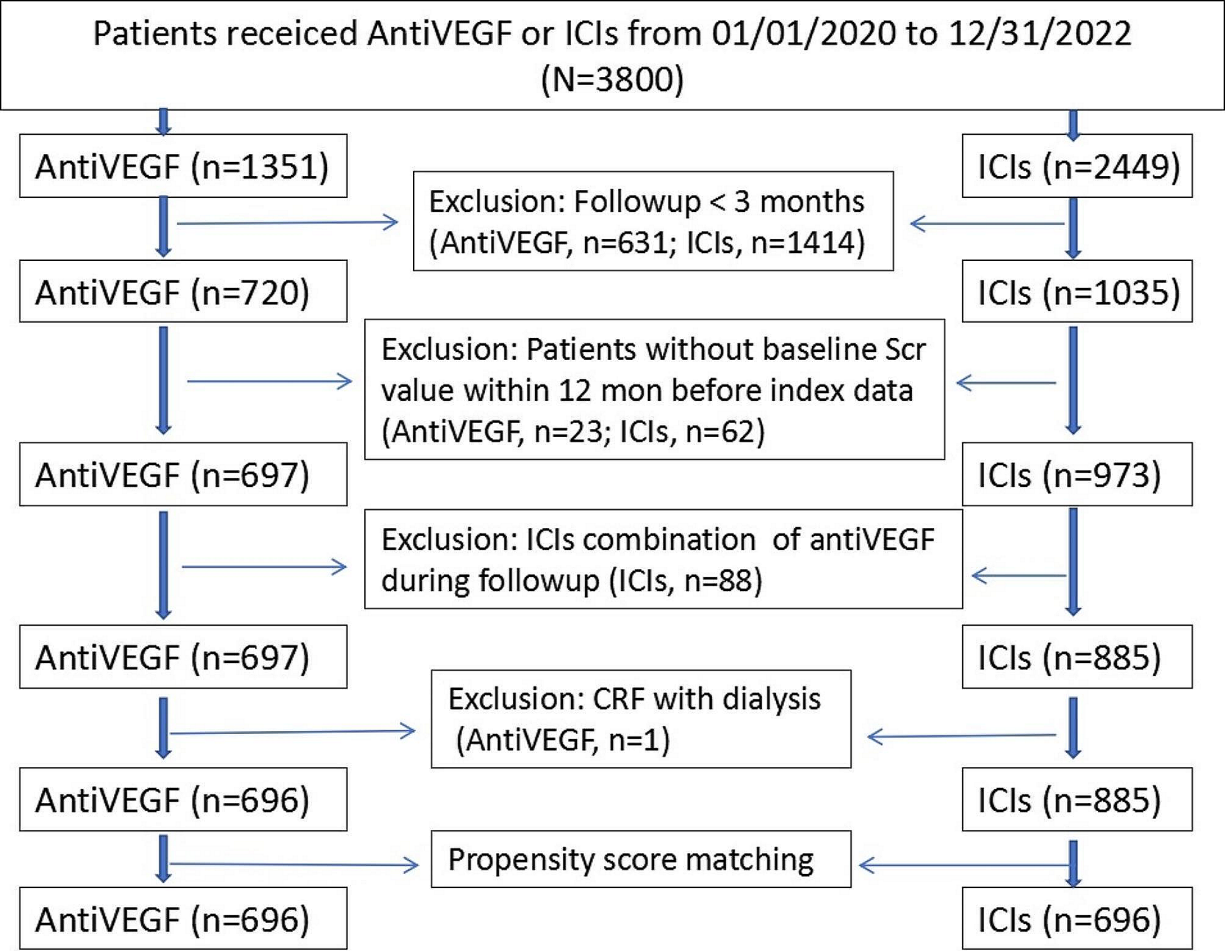
Description of patients’ selection. AntiVEGF: anti-vascular endothelial growth factor monoclonal antibody; ICIs, immune checkpoint inhibitors; CRF, chronic renal failure

Between Jan 2020 and December 2022, 1351 and 2449 cancer patients initiated anti-VEGF and ICIs, respectively. Eventually, 1581 patients were enrolled, of whom 696 and 885 patients received anti-VEGF and ICIs respectively. After propensity score matching, there were 696 patients in the ICIs group included in the analysis (Fig. 1). The most common cancers included lung, digestive and genitourinary cancers. Hypertension and infection were the most frequent comorbidities in the two cohorts.

After propensity score matching, the two groups were well balanced with SMDs less than 0.20 for most main clinical characteristics (Table 1). There were no significant differences in cancer stages between the anti-VEGF group and the ICIs group before and after PS matching (Supplementary Table 1).

**Table 1:** Baseline characteristics before and after PS matching

Characteristic	antiVEGF	Before PS Matching			After PS Matching			
		ICIs	Overall	SMD	ICIs	Overall	SMD	
*N*	696	885	1581		696	1392		
Male, *n*(%)	350 (50.3)	732 (82.7)	1082 (68.4)	0.731	563 (80.9)	913 (65.6)	0.68	
Age < 60 year, *n*(%)	264 (37.9)	299 (33.8)	563 (35.6)	0.087	258 (37.1)	522 (37.5)	0.018	
age, mean (SD)	62.0 (10.3)	63.0 (11.2)	62.6 (10.8)	0.094	62.2 (11.2)	62.1 (10.77)	0.022	
Cancer category, *n*(%)				0.453			0.489	
Lung	233 (33.5)	323 (36.5)	556 (35.2)		248 (35.6)	481 (34.6)		
Digestive system	350 (50.3)	332 (37.5)	682 (43.1)		264 (37.9)	614 (44.1)		
Genito-urinary system	83 (11.9)	81 (9.2)	164 (10.4)		56 (8.0)	139 (10.0)		
others	30 (4.3)	149 (16.8)	179 (11.3)		128 (18.4)	158 (11.4)		
WBC, median [IQR]	5.8 [4.6, 7.1]	6.10 [4.60, 7.70]	5.9 [4.6, 7.5]	0.144	6.1 [4.7, 7.6]	5.9 [4.6, 7.4]	0.138	
Hb, mean (SD)	121 (18)	121 (20)	121 (19)	0.028	122(20)	122(19)	0.054	
PLB, median [IQR]	216 [171, 275]	215 [162, 284]	216[166, 280]	0.018	213 [162, 279]	215 [166, 278]	0.038	
SALB < 35 g/l, *n*(%)	99 (14.2)	186 (21.0)	285 (18.0)	0.179	101 (14.5)	200 (14.4)	0.008	
SALB, median [IQR]	40.3 [37.0, 43.0]	38.7 [35.4, 41.7]	39.4 [36.1, 42.3]	0.309	39.6 [36.6, 42.3]	39.9 [36.8, 42.8]	0.108	
Scr, median [IQR]	68.0 [56.0, 81.0]	72.0 [60.0, 85.0]	70.0 [58.0, 84.0]	0.199	70.0 [59.0, 84.0]	69.0 [57.0, 82.0]	0.091	
CKD, n(%)	90 (12.9)	169 (19.1)	259 (16.4)	0.169	115 (16.5)	205 (14.7)	0.101	
Hypertension, *n*(%)	283 (40.7)	323 (36.5)	606 (38.3)	0.086	246 (35.3)	529 (38.0)	0.11	
Diabetes, *n*(%)	129 (18.5)	173 (19.5)	302 (19.1)	0.026	126 (18.1)	255 (18.3)	0.011	
Infection, *n*(%)	183 (26.3)	279 (31.5)	462 (29.2)	0.116	214 (30.7)	397 (28.5)	0.099	
Hypovolume, *n*(%)	3 (0.4)	12 (1.4)	15 (0.9)	0.098	10 (1.4)	13 (0.9)	0.105	
Medications								
Platinum, *n*(%)	510 (73.3)	707 (79.9)	1217 (77.0)	0.157	552 (79.3)	1062 (76.3)	0.142	
Paclitaxel, *n*(%)	114 (16.4)	312 (35.3)	426 (26.9)	0.442	249 (35.8)	363 (26.1)	0.453	
Docetaxel, *n*(%)	41 (5.9)	41 (4.6)	82 (5.2)	0.056	29 (4.2)	70 (5.0)	0.079	
Capecitabine, *n*(%)	181 (26.0)	24 (2.7)	205 (13.0)	0.704	20 (2.9)	201 (14.4)	0.697	
Pemetrexed, *n*(%)	150 (21.6)	84 (9.5)	234 (14.8)	0.338	61 (8.8)	211 (15.2)	0.362	
Irinotecan, *n*(%)	228 (32.8)	24 (2.7)	252 (15.9)	0.856	18 (2.6)	246 (17.7)	0.861	
Etoposide, *n*(%)	13 (1.9)	31 (3.5)	44 (2.8)	0.101	25 (3.6)	38 (2.7)	0.106	
COX-2i, *n*(%)	180 (25.9)	177 (20.0)	357 (22.6)	0.14	140 (20.1)	320 (23.0)	0.137	
NSAIDs, *n*(%)	77 (11.1)	131 (14.8)	208 (13.2)	0.112	101 (14.5)	178 (12.8)	0.103	
Steriods, *n*(%)	232 (33.3)	312 (35.3)	544 (34.4)	0.04	241 (34.6)	473 (34.0)	0.027	
Diuretics, n*n*(%)	68 (9.8)	80 (9.0)	148 (9.4)	0.025	52 (7.5)	120 (8.6)	0.082	
RASi, *n*(%)	101 (14.5)	159 (18.0)	260 (16.4)	0.094	129 (18.5)	230 (16.5)	0.108	
Antibiotics, *n*(%)	20 (2.9)	46 (5.2)	66 (4.2)	0.118	37 (5.3)	57 (4.1)	0.123	

Anti-VEGF: anti-vascular endothelial growth factor monoclonal antibody; ICIs, immune checkpoint inhibitors; PS, propensity score; WBC, white blood cell; Salb, serum albumin; Scr, serum creatinine; CKD, chronic kidney disease; COX-2i, cyclooxygenase-2 inhibitors; COX-1i, cyclooxygenase-1 inhibitors; ACEI, angiotensin-converting enzyme inhibitor; ARB, angiotensin receptor blocker; $, Antibiotics include compound sulfamethoxazole, vancomycin and aminogly cosides

### Primary outcome ITT analyses

**Fig. 2:**
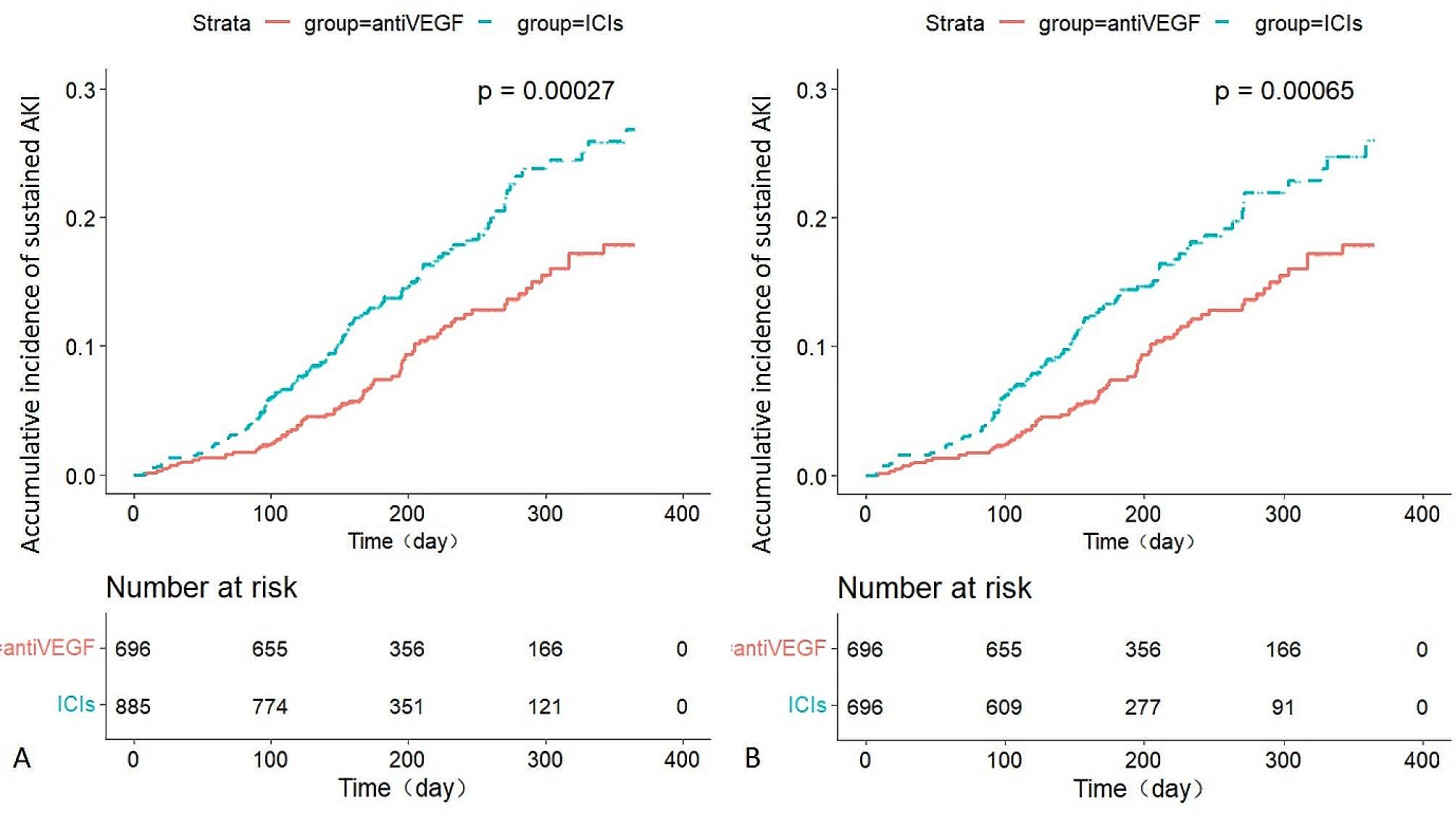
Cumulative incidence of sustained AKI for patients receiving anti-VEGF vs. ICIs. Kaplan-Meier curves depicted for patients with anti-VEGF during one year (red line) versus those with ICIs (green line). AKI, acute kidney injury; AntiVEGF: anti-vascular endothelial growth factor monoclonal antibody; ICIs, immune checkpoint inhibitors

**Fig. 3:**
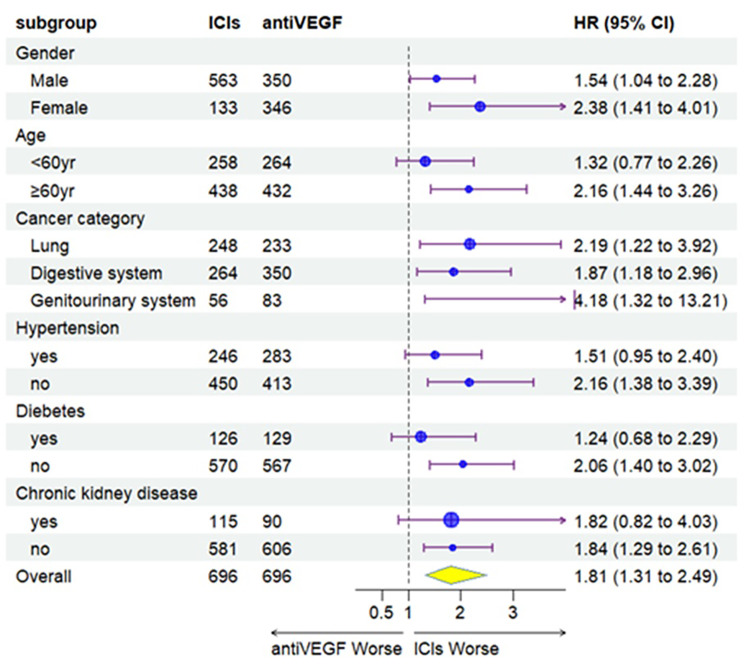
The association between initiation of ICIs, compared with anti-VEGF, and sustained AKI overall and by subgroups after propensity score matching. AntiVEGF: anti-vascular endothelial growth factor monoclonal antibody; ICIs, immune checkpoint inhibitors

A total of 206 (13.0%) cancer patients developed sustained AKI following the initiation of anti-VEGF or ICIs therapy. Before propensity score matching, the respective event rates of sustained AKI were 10.6% vs. 14.9% in cancer patients receiving anti-VEGF vs. ICIs, most of which were AKI-1 stage (8.2% vs. 12.7%). After propensity score matching, there were 103 sustained AKI episodes (14.7%) in patients receiving ICIs, the majority being of mild severity (AKI-1 stage:12.2%) (Table 2). Compared with the anti-VEGF group, the ICIs group had a higher cumulative incidence of sustained AKI in one year before PS matching (26.8% vs. 17.8%, *P* < 0.001) or after PS matching (26.1% vs. 17.8%, *P* < 0.001) (Fig. 2). ICIs therapy was associated with an increased risk of sustained AKI events in the entire population (HR 2.0; 95%CI 1.3 to 2.5; *P* = 0.001) and especially in those with genitourinary cancer (HR 4.2; 95%CI 1.3 to 13.2; *P* = 0.015) after propensity score matching (Fig. 3). Similar findings were observed before propensity score matching, where the HR was 1.7 (95% CI 1.3–2.3, *P* < 0.001) and 4.3 (95%CI 1.4–12.9, *P* = 0.009) for the overall population or those with genitourinary cancer, respectively (Supplementary Fig. 1). Compared with patients receiving anti-VEGF, patients receiving Pembrolizumab, Sintilimab, Toripalimab, Camrelizumab, or Tislelizumab had more sustained AKI events in one year (Supplementary Fig. 2).

**Table 2:** Characteristics of AKI events before and after PS matching

Characteristics	antiVEGF	Before PS matching			After PS matching	
		ICIs	Overall		ICIs	Overall
*N*	696	885	1581		696	1392
Sustained AKI, *n* (%)	74(10.6)	132(14.9)	206(13.0)		103(14.7)	177(12.7)
stage 1, *n* (%)	57(8.2)	112(12.7)	169(10.7)		85(12.2)	142(10.2)
stage 2, *n* (%)	7(1.0)	5(0.6)	12(0.7)		5(0.7)	12(0.9)
stage 3, *n* (%)	10(1.4)	15(1.7)	25(1.6)		13(1.9)	23(1.7)
Non-sustained AKI, *n* (%)	12(1.7)	112(12.7)	124(7.8)		83(11.9)	95(6.8)

Anti-VEGF: anti-vascular endothelial growth factor monoclonal antibody; ICIs, immune checkpoint inhibitors; PS, propensity score

### Risk factors for sustained AKI by multivariable cox regression

In patients receiving anti-VEGF, sustained AKI was associated with age > 60 year (HR 0.5; 95%CI 0.3 to 0.8; *P* = 0.007), type of cancer (HR 1.5; 95%CI 1.1 to 2.1 *P* = 0.013), baseline Salb > 35 g/l (HR 0.5; 95%CI 0.3 to 0.9; *P* = 0.027), diagnosis of pre-existing diabetes (HR 2.4; 95%CI 1.4 to 4.1; *P* = 0.001) and the presence of infection (HR 2.1; 95%CI 1.2 to 3.5; *P* = 0.006). Use of Pemetrexed (HR 2.1; 95%CI 1.0 to 4.5; *P* = 0.049) and diuretics (HR 2.5; 95%CI 1.4 to 4.6; *P* = 0.003) were also associated with sustained AKI. In patients receiving ICIs, sustained AKI was associated only with male (HR 1.7; 95%CI 1.1 to 2.7; *P* = 0.029), baseline Salb > 35 g/l (HR 0.3; 95%CI 0.2 to 0.5; *P* < 0.001) and use of nephrotoxic antibiotics (HR 2.5; 95%CI 1.2 to 5.5; *P* = 0.019) after propensity score matching (Table 3).

**Table 3:** Risk factors for AKI in patients receiving anti-VEGF or ICIs

	Anti-VEGF					ICIs patients								
	Before or after PS matching					Before PS matching					After PS matching			
Variable	**HR**	**95% CI**		**P**		**HR**	**95% CI**		*P*		**HR**	**95% CI**		*P*
Male	1.0	0.6	1.7	0.989		1.8	1.2	2.7	0.007		1.7	1.1	2.7	0.029
Age > 60yr	0.5	0.3	0.8	0.007		1.2	0.8	1.8	0.466		1.3	0.8	2.1	0.243
Cancer category	1.5	1.1	2.1	0.013		1.0	0.8	1.3	0.822		1.0	0.8	1.3	0.784
Salb > 35 g/l	0.5	0.3	0.9	0.027		0.5	0.3	0.7	0.000		0.3	0.2	0.5	0.000
CKD	0.8	0.4	1.5	0.438		1.0	0.7	1.6	0.876		1.0	0.6	1.8	0.922
HP	1.0	0.6	1.8	0.921		1.5	1.0	2.2	0.049		1.3	0.8	2.0	0.246
DM	2.4	1.4	4.1	0.001		1.1	0.7	1.7	0.794		1.2	0.7	2.1	0.450
Infection	2.1	1.2	3.5	0.006		0.9	0.6	1.3	0.564		0.8	0.5	1.4	0.487
Platinum	1.4	0.7	2.6	0.333		1.1	0.7	1.8	0.722		0.9	0.5	1.6	0.701
Paclitaxel	0.5	0.2	1.1	0.091		1.0	0.6	1.5	0.888		1.2	0.8	1.9	0.432
Capecitabine	1.2	0.7	2.3	0.519		0.5	0.1	2.3	0.414		0.4	0.1	3.1	0.395
Pemetrexed	2.1	1.0	4.5	0.049		1.0	0.5	1.8	0.872		1.3	0.6	2.6	0.521
Irinotecan	0.7	0.4	1.4	0.322		0.8	0.2	3.3	0.753		1.2	0.3	5.1	0.822
COX2i	1.1	0.6	2.0	0.701		1.1	0.7	1.7	0.655		1.0	0.6	1.6	0.900
NSAIDs	1.3	0.6	2.6	0.454		1.3	0.9	2.1	0.212		1.5	0.9	2.4	0.130
Steriods	0.9	0.5	1.5	0.584		1.3	0.9	2.0	0.196		1.2	0.8	2.0	0.367
Diuretics	2.5	1.4	4.6	0.003		1.6	0.9	2.6	0.089		1.4	0.7	2.7	0.327
RASi	1.4	0.7	2.7	0.302		0.6	0.4	1.1	0.092		0.7	0.4	1.3	0.237
Antibiotics	0.2	0.0	1.5	0.109		2.0	1.0	3.8	0.045		2.5	1.2	5.5	0.019

Anti-VEGF: anti-vascular endothelial growth factor monoclonal antibody; ICIs, immune checkpoint inhibitors; Salb, serum albumin; CKD, chronic kidney disease; COX-2i, cyclooxygenase-2 inhibitors; COX-1i, cyclooxygenase-1 inhibitors; ACEI, angiotensin-converting enzyme inhibitor; ARB, angiotensin receptor blocker; $, Antibiotics include compound sulfamethoxazole, vancomycin and aminoglycosides

## Discussion

In this retrospective cohort of cancer patients with follow-up more than three months, initiation of ICIs was associated with significantly higher risks of sustained AKI than those with anti-VEGF. To our knowledge, this is the first real-world study exploring AKI events of the inpatient with malignancies receiving anti-VEGF vs. ICIs. The efficacy and safety of anti-VEGF and ICIs have been reported in many clinical trial [1–5, 12–14]. However, no previous study was designed with sustained AKI as the primary end point. a Danish study of 37,267 incident cases of cancer showed that the 1-year risk of AKI was 17.5% [15]. Colorectal cancer survivors were at increased risk of AKI for several years after cancer diagnosis [16]. AKI occurs in up to 31.8-66.5% of patients with hematologic cancers [17–19]. In our study, most patients receiving anti-VEGF or ICIs had lung cancer, digestive system and genitourinary system cancer. Cumulative incidence of sustained AKI in one year was 17.8% in the anti-VEGF group, which was similar with the one-year incidence of AKI (19.2%) in patients with metastatic colorectal cancer treated with chemotherapy combined with bevacizumab [14]. The incidence of AKI with ICIs has been reported to be as low as 2 -4.5% from the results of cancer trials and up to 17-18.2% reported in emerging data [7, 20–25]. In our study, the incidence of sustained AKI was about 15% and accumulative incidence in one year was 26.8% in the ICIs group.

Results of our subgroup analyses suggested a possible lack of significant difference of sustained AKI in younger patients (aged < 60 years) and those who had hypertension, diabetes or chronic kidney disease, which would support prioritizing the prescription of anti-VEGF to older people and those without hypertension, diabetes or chronic kidney disease. There is increasing evidence showing that anti-VEGF treatment is associated with cases of accelerated hypertension, worsening proteinuria, glomerular disease, thrombotic microangiopathy, and possible renal function decline [8, 10]. Meta-Analysis has suggested intravitreal use of anti-VEGF was not associated with an AKI risk [26]. Further comparative research on the AKI events in cancer patient receiving anti-VEGF vs. ICIs is needed, as our results could be confounded by the retrospective data.

Our study showed there were dramatic differences in the risk factors for sustained AKI between the anti-VEGF group and the ICIs group except that serum albumin level was a strong risk factor for sustained AKI in both groups. Our previous study demonstrated that serum albumin level was associated with the incidence of AKI in idiopathic nephrotic syndrome [27]. It may have important treatment implications to correct the presenting hypoalbuminemia during anti-VEGF or ICIs therapy in cancer patients.

In the anti-VEGF group, other baseline characteristics except for serum albumin, such as age, malignancy type, diabetes, infection, pemetrexed treatment or use of diuretics, were associated with sustained AKI. Randomized clinical trial also have demonstrate that pemetrexed plus anti-VEGF therapy had high toxicities but no survival benefit [28]. Guideline recommended that anti-VEGF therapy should not be added to pemetrexed and platinum therapy or given as maintenance. Previous studies have reported that use of diuretics was a risk factor of AKI in cancer patients receiving ICIs [29, 30]. An association between diuretics use and sustained AKI was observed in the anti-VEGF group, but not in the ICIs group in our study. The small proportion of individuals receiving diuretics in the ICIs group may have precluded detection of such an association. As in previous study [7], we did not find a statistically significant association between sustained AKI events and the presenting of chronic kidney disease in the two groups. Therefore, the use of anti-VEGF or ICIs should not be withheld in cancer patients with chronic kidney disease. Cancer patients are at risk for AKI that is caused by sepsis, direct kidney injury due to the primary cancer, infection, the nephrotoxic effects of anticancer therapies, or metabolic disturbances such as tumor lysis syndrome and hypercalcemia [19]. The different risk factors for AKI between the two groups suggested that a careful evaluation of patients’ comorbidity and combined drug therapy is needed to prevent AKI.

Our study has several important limitations. First, it was a retrospective study. We only included patients who had at least three months follow-up period to ensure that patients receiving the majority of their care outside our health care system were not included in the analysis, which will result in an underestimation of AKI frequency. Second, although the cohort is large, it was sourced from a single center and was a predominantly Chinese population, raising concerns about the generalizability to other populations. Third, most of the AKI episodes were neither diagnosed nor treated by nephrologists. As such, the definite cause of AKI could not be ascertained in all cases. Furthermore, AKI in patients with cancer has diverse causes and multiple mechanisms. Establishing ICIs or anti-VEGF related nephrotoxicity versus co-prescription of other drugs associated with AIN (e.g., antibiotics and NSAIDs) may be challenging. Only a minority of ICIs or anti-VEGF related nephrotoxicity was confirmed by renal biopsy. As a result, we were able to evaluate overall sustained AKI events, but not necessarily events specific to ICIs or anti-VEGF nephrotoxicity. Forth, the ability to confirm the timeline and acuity of sustained AKI precisely may have been affected in the patients who were not admitted to the hospital because laboratory tests were not performed daily for outpatients. Fifth, we did not provide evidence that AKI in the anti-VEGF group was recovered by drug withdrawal and that AKI in the ICIs group was recovered by steroid administration. Sixth, the frequency of immune-related adverse events that contributes to the emergence of the difference in AKI between the two group was not explicitly stated in our study.

In conclusion, this retrospective cohort study of cancer patients receiving anti-VEGF vs. ICIs showed that initiation of ICIs was associated with significant increase in risk of sustained AKI. Serum albumin was a major risk factor for AKI in these patients. A careful evaluation must be performed to prevent AKI because there were different risk factors between anti-VEGF and ICIs treatment.

### Electronic supplementary material

Below is the link to the electronic supplementary material.

Supplementary Material 1

Supplementary Material 2

Supplementary Material 3

## Data Availability

The datasets used during the current study available from the corresponding author on reasonable request.

## Abbreviations

AntiVEGF: Anti vascular endothelial growth factor monoclonal antibody
ICIs: Immune checkpoint inhibitors
AKI: Acute kidney injury
PD-1: Programmed cell death-1
PD-L1: Programmed cell death-ligand 1
HIS: Hospital information system
SMD: Standardized mean difference
KDIGO: Kidney Disease Improving Global Outcomes
Scr: Serum creatinine
