# Supplementary material for: Acute kidney injury in cancer patients receiving anti-vascular endothelial growth factor monoclonal antibody vs. immune checkpoint inhibitors: a retrospective real-world study
Source: BMC Cancer. 2024 Jun 24;24:756. doi: 10.1186/s12885-024-12540-y (PMC11194933; doi:10.1186/s12885-024-12540-y)
Supplement: Supplementary file 1 — Supplementary Material 1 [file 12885_2024_12540_MOESM1_ESM.docx]

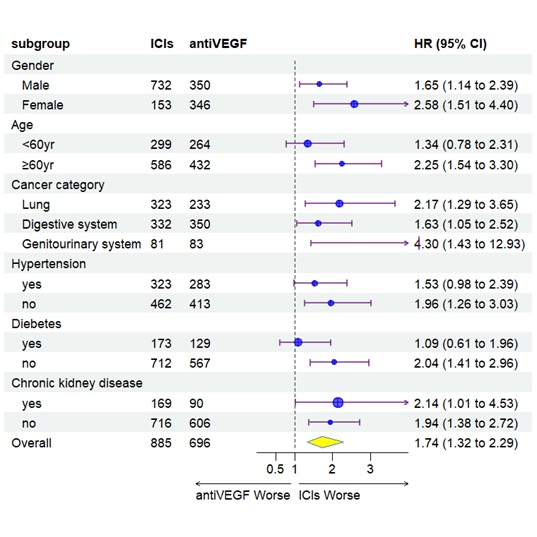


Supplementary figure 1. The association between initiation of ICIs, compared with antiVEGF, and sustained AKI overall and by subgroups before PS matching.
