# Supplementary material for: Acute kidney injury in cancer patients receiving anti-vascular endothelial growth factor monoclonal antibody vs. immune checkpoint inhibitors: a retrospective real-world study
Source: BMC Cancer. 2024 Jun 24;24:756. doi: 10.1186/s12885-024-12540-y (PMC11194933; doi:10.1186/s12885-024-12540-y)
Supplement: Supplementary file 2 — Supplementary Material 2 [file 12885_2024_12540_MOESM2_ESM.docx]

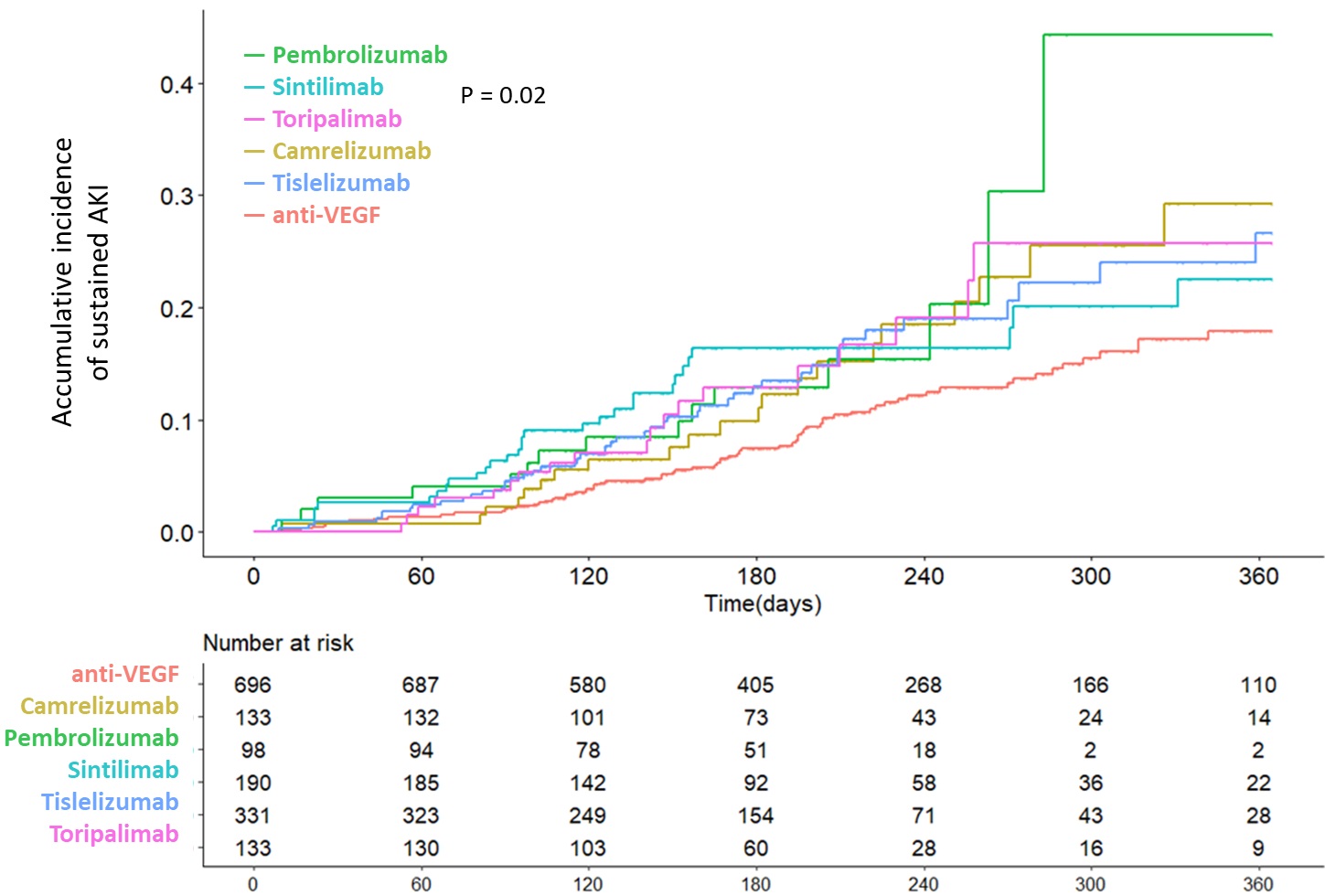


Supplementary figure 2. accumulative incidence of sustained AKI for patients receiving Anti-VEGF vs specific ICIs. Pembrolizumab vs anti-VEGF, P=0.023; Pembrolizumab vs Sintilimab, P=0.834; Pembrolizumab vs Toripalimab, P=0.759; Pembrolizumab vs Camrelizumab, P=0.571; Pembrolizumab vs Tislelizumab, P=0.680; Sintilimab vs anti-VEGF, P=0.014; Sintilimab vs Toripalimab, P=0.882; Sintilimab vs Camrelizumab, P=0.917; Sintilimab vs Tislelizumab, P=0.814; Toripalimab vs anti-VEGF, P=0.038; Toripalimab vs Camrelizumab, P=0.874; Toripalimab vs Tislelizumab, P=0.931; Camrelizumab vs anti-VEGF, P=0.036; Camrelizumab vs Tislelizumab, P=0.998; Tislelizumab vs anti-VEGF, P=0.007. AKI, acute kidney injury; Anti-VEGF: anti-vascular endothelial growth factor monoclonal antibody; ICIs, immune checkpoint inhibitors.
