# Supplementary material for: Acute kidney injury in cancer patients receiving anti-vascular endothelial growth factor monoclonal antibody vs. immune checkpoint inhibitors: a retrospective real-world study
Source: BMC Cancer. 2024 Jun 24;24:756. doi: 10.1186/s12885-024-12540-y (PMC11194933; doi:10.1186/s12885-024-12540-y)
Supplement: Supplementary file 3 — Supplementary Material 3 [file 12885_2024_12540_MOESM3_ESM.docx]

Supplementary table 1. Comparison of cancer stage between anti-VEGF group and ICIs

before and after PS matching

| **Cancer stage** |  | Before PS matching | | After PS matching | |
| --- | --- | --- | --- | --- | --- |
|  | Anti-VEGF | ICIs | P | ICIs | P |
| N | 696 | 885 |  | 696 |  |
| Overall，n(%) |  |  |  |  |  |
| II stage | 7(1.0) | 12(1.4) | 0.815 | 9(1.3) | 0.803 |
| III stage | 71(10.2) | 91(10.3) |  | 66(9.5) |  |
| IV stage | 618(88.8) | 782(88.3) |  | 621(89.2) |  |
| Cancer category |  |  |  |  |  |
| Lung，n(%) |  |  |  |  |  |
| II stage | 6(2.6) | 7(2.2) | 0.556 | 5(2.0) | 0.583 |
| III stage | 59(25.3) | 70(21.7) |  | 53(21.4) |  |
| IV stage | 168(72.1) | 246(76.2) |  | 190(76.6) |  |
| Digestive system，n(%) |  |  |  |  |  |
| II stage | 1 (0.3) | 0 (0.0) | 0.621 | 0 (0.0) | 0.290 |
| III stage | 3(0.9) | 3(0.9) |  | 1(0.4) |  |
| IV stage | 346 (98.9) | 329 (99.1) |  | 263 (99.6) |  |
| Genito-urinary system，n(%) |  |  |  |  |  |
| II stage | 0 (0.0) | 2 (2.5) | 0.122 | 2 (3.6) | 0.072 |
| III stage | 9(10.8) | 15 (18.5) |  | 10 (17.9) |  |
| IV stage | 74 (89.2) | 64 (79.0) |  | 44 (78.6) |  |
| others，n(%) |  |  |  |  |  |
| II stage | 0 (0.0) | 3 (2.0) | 0.535 | 2 (1.6) | 0.618 |
| III stage | 0 (0.0) | 3 (2.0) |  | 2 (1.6) |  |
| IV stage | 30 (100) | 143 (96.0) |  | 124 (96.8) |  |
